# 

**Published:** 1880-08-20

**Authors:** 


☞ Subscribers in arrears are requested to remit at once. We desire our friends not to send money to third parties for us, if it can be avoided. The mail is the best and safest medium and gives least trouble to all parties.

### *RECEIPTED.*

[Receipts not acknowledged privately are entered here.]

1880.—Drs. P. S. Anderson; W. H. Wilson; E. W. Lane; Robert James; A. F. Sanders; I. F. Mooty; M. Moore; M. S. Posey; F. P. H. Akers; A. W. Rickman; B. E. Clark; G. W. Earle; S. C. Eve; Thos. S. Mitchell; I. H. Goss; Thos. J. Hendley.

